# The impact of smoking on adherence to treatment for latent tuberculosis infection

**DOI:** 10.1186/1471-2458-6-66

**Published:** 2006-03-14

**Authors:** Mélanie Lavigne, Isabelle Rocher, Colin Steensma, Paul Brassard

**Affiliations:** 1 McGill University Health Center – Division of Clinical Epidemiology – Royal Victoria Hospital, Montreal, Canada; 2 McGill University Health Center – Montreal Chest Institute, Montreal, Canada

## Abstract

**Background:**

Studies have shown an association between smoking and tuberculosis (TB) infection, disease and TB-related mortality. We hypothesized that smokers with latent tuberculosis infection (LTBI) are less likely to comply with their LTBI treatment regimen, thus increasing their risk of developing active disease. We thus documented the impact of smoking on adherence to LTBI treatment.

**Method:**

Between 1998 and 2000, a convenience sample of patients undergoing treatment for LTBI completed a questionnaire on smoking status. Level of adherence to LTBI treatment was tested for associations with socio-demographic profile, and smoking status

**Results:**

320 patients were recruited, and 302 (94%) completed the questionnaire. Smoking prevalence was 21%. 72% of patients were adherent to LTBI treatment. Women (OR = 2.0; 95% CI: 1.2–3.3) and non-smokers (OR = 1.8; 95% CI: 1.0–3.3) were associated with adherence to LTBI treatment. Only gender was found as an independent predictor of adherence after adjusting for age and smoking status (OR = 1.9; 95% CI: 1.06–3.3).

**Conclusion:**

Males and smokers need to have extra supervision to ensure compliance with LTBI treatment.

## Background

Studies have shown an association between smoking and tuberculosis (TB) infection,[1] disease, [2-6] and TB-related mortality. [7] Based on the premise that smokers tend to be less compliant to medication [8,9] we hypothesized that smokers with latent tuberculosis infection (LTBI) are less likely to comply with their LTBI treatment regimen, thus increasing their risk of developing active disease. We thus set out to document the impact of smoking on adherence to LTBI.

## Methods

For the 1998–2000 period, a convenience sample was taken of consecutive patients initiating treatment for LTBI who were given the opportunity to undergo a self-administered questionnaire (French-English) on baseline demographic characteristics. Patients were recruited at a specialized TB clinic located at the Montreal Chest Institute (MCI), which deals mainly but not exclusively with new adult immigrants and refugee claimants for whom immigration screening evaluation suggested inactive TB. [10] Eligibility of patients was defined according to their level of understanding of the languages of the survey. A written informed consent to participate in the study was obtained before administration of the questionnaire.

Smoking status and nicotine dependence was assessed by the nurse clinician. We used the Canadian tobacco use monitoring survey (CTUMS) terminology to define current and non- and ex- smokers. [11] Nicotine dependence in smokers was assessed using a 10 point visual scale, with 1 denoting minimum dependence and 10 denoting maximum, using a modified Fagerström test for nicotine dependence. [12] Subjects were classified as nicotine dependent if the score was 6 or higher.

The medical evaluation followed the Canadian Thoracic Society Guidelines for tuberculosis control [13] and included sputum smear and cultures when indicated. Adherence to therapy was assessed by the clinic nurses through pill counts, patient self-report and general attendance at the scheduled medical visits. Adherence was considered adequate if patients took more than 80% of total prescribed doses for the LTBI treatment regimen, with the remainder being considered non adherent.

Level of adherence to LTBI treatment was tested for associations with socio-demographic profile (age, gender, region of birth), and smoking status (current/non- and ex-smokers). Univariate analysis using chi-square test, Fisher's exact test or t-test was used to compare various characteristics of interest to LTBI treatment adherence. Significance was defined at the p <= 0.05 level. We performed a multiple logistic regression analysis using a step-wise approach to estimate the independent contribution of significant characteristics found in the univariate analysis towards adherence to LTBI. Data analysis was done using the SAS program (version 8.2). Ethical approval was obtained from the Montreal Chest Institute ethics committee.

## Results

337 patients were initially asked to participate and 320 were recruited (180 males) with a mean age of 35.5 (SD ± 10.8) years old. We obtained adherence status in 302/320 (94%) participants. More than 87% (281/320) of our study group was found to be foreign born and 64% of the latter had immigrated to Canada within the previous 5 years. Most of our participants (33%) came from the Indian sub continent (mostly India and Pakistan) and 17% from North Africa (mostly Algeria and Morocco). Smoking prevalence was 21% (28% and 20% among Canadian born and foreign born respectively). The overall mean nicotine dependence among smokers was low (2.9/10 (SD ± 2.4); median 3.0) and reflected the fact that only 17% of current smokers reported being tobacco dependent (Table). Smokers smoked in general less than 12 cigarettes per day (48%), while 23% smoked at least 25 cigarettes a day (the equivalent of a half pack and a full pack of cigarettes respectively). Overall, adequate adherence to LTBI treatment was found in 217/302 (72%) participants. In the univariate analysis, factors associated with adherence to LTBI treatment included being female (odds ratio (OR) = 2.0; 95% confidence interval (CI): 1.2–3.3) and being a non-smoker (OR = 1.8; 95% CI: 1.0–3.3). Characteristics such as age, region of birth, and nicotine dependence among smokers were not associated with adherence to LTBI (Table). In the multiple logistic regression analysis, only being female (OR = 1.9; 95% CI: 1.1–3.3) was independently associated with adherence when adjusted for age and smoking status.

## Discussion

In this study, smoking prevalence was found among 21% of our study population and was more prevalent in men (33%) than in women (6%). Our finding is comparable to the prevalence found in the respective region of birth of the participants. [14] As 88% of our study group were from the developing world, it was not surprising to see that men were more likely to smoke than woman. [15,16]

Adequate adherence to LTBI was found in 72% of patients and is consistent with previous studies done at the MCI. [10] In our univariate analysis, men and smokers were less likely to be compliant to treatment. The multiple logistic regression analysis showed that gender was the only independent predictor of adherence. Males may be less compliant to LTBI treatment for economic reasons. In various cultures, men are the main contributors to the family income and cannot afford to take time out for a medical visit to a clinic. Indeed, travel time to and from the clinic represents time absent from work and potentially less money earned. [17] This speculation is supported by the finding among our study group that men were less likely than women to report attending regular medical visits (p < 0.001). Another plausible reason would be that males are less likely to adhere to healthy behaviours and prevention messages such as smoking cessation and adherence to treatment.

Certain limitations in this study have to be recognized. The self-report of smoking status could have been biased by a certain degree of social desirability from the respondent which may have underestimated the true prevalence of smoking. Characteristics such as economic status and language barriers/comprehension (although the latter was initially addressed in the participant selection) might have also influenced adherence, especially in the foreign-born, but were not sought after in this study. Our convenience sample of participants was nonetheless representative of the overall MCI clinic attendance with respect to basic socio-demographic factors,[18,19] although generalizability of our findings to other clinical sites is somewhat limited. We also had a small proportion (5%) of eligible subjects who refused to participate. We were not able to characterize these individuals but there is no reason to think that they were any different from our study group regarding the variables of interest.

## Conclusion

Overall, our findings indicate that males and smokers need to have extra supervision to ensure compliance with LTBI treatment.

## Abbreviations

TB-tuberculosis

LTBI-latent tuberculosis infection

MCI-Montreal Chest Institute

CTUMS-Canadian tobacco use monitoring survey

OR-odds ratio

CI-confidence interval

SD-standard deviation

## Competing interests

The author(s) declare that they have no competing interests.

## Authors' contributions

ML participated in the design, collected and updated data, performed part of the statistical analysis and drafted the manuscript. IR conceived the study, and participated in its design and helped to draft the manuscript. CS performed part of the statistical analysis and helped to draft the manuscript. PB supervised and insured coordination of the study and helped to draft the manuscript. All authors read and approved the final manuscript.

## Pre-publication history

The pre-publication history for this paper can be accessed here:



## Figures and Tables

**Table 1:** Patient characteristics associated with LTBI treatment adherence.

**Characteristics**	**Adherence**		
	
	**Non adherent N = 85(%)**	**Adequate N = 217(%)**	**p-value**
	
**Gender**			
Male	58 (68)	113 (52)	0.01
Female	27 (32)	104 (48)	
**Age in years**			
15–30	35 (41)	78 (36)	
31–45	39 (46)	97 (45)	0.4
> 45	11 (13)	42 (19)	
Mean age (± S.D.)	35 (± 10)	36 (± 11)	0.8
**Region of origin**			
Canadian born	11 (13)	26 (12)	0.8
Foreign born	74 (87)	191 (88)	
Africa	21 (25)	58 (27)	
Asia	33 (39)	85 (39)	
Europe	4 (5)	16 (8)	
Latin America	16 (18)	30 (14)	
**Smoking status**			
**Current smoker**	25 (29)	40 (18)	0.04
**Non/ex-smoker**	60 (71)	177 (82)	
Nicotine dependence*†			
< 6	10 (77)	15 (88)	0.6
≥ 6	3 (23)	2 (12)	

*: Modified Fagerström test scores among smokers only † n = 30

SD: Standard deviation
